# The complete mitochondrial genome of *Sarcophaga brevicornis* (Diptera: Sarcophagidae)

**DOI:** 10.1080/23802359.2019.1644561

**Published:** 2019-07-24

**Authors:** Changquan Zhang, Wang Shiwen, Yanjie Shang, Xiao Shen, Yadong Guo

**Affiliations:** a Institute of Reproductive and Stem Cell Engineering, School of Basic Medical Sciences, Central South University, Changsha, China;; b Department of Forensic Science, School of Basic Medical Sciences, Xinjiang Medical University, Ürümqi, China;; c Department of Forensic Science, School of Basic Medical Sciences, Central South University, Changsha, China

**Keywords:** Mitogenome, *Sarcophaga brevicornis*, phylogenetic analysis

## Abstract

*Sarcophaga brevicornis* (Diptera: Sarcophagidae) was of utmost forensic importance due to their wide distribution, ubiquitous, and synanthropic nature. The complete mitochondrial genome (mitogenome) of *S. brevicornis* was first sequenced and assembled in this study. The length of circular mitogenome was 15,152 bp, which showed in a typical arthropod genome, including 13 protein-coding genes (13 PCGs), two ribosomal RNA (two rRNA) genes, 22 transfer RNA (22 tRNA) genes, and an AT-rich region. Its nucleotide composition was A 39.0%, C 12.7%, G 10.6%, and T 37.7%. Furthermore, phylogenetic relationships of *S. brevicornis* and the published sarcophagid species were evaluated based on 13 PCGs. The results indicated that *S. brevicornis* was clearly separated from the other sarcophagid species, but it was closed to the species of *Sarcophaga similis*. This study provided a significant database reference for genetic structure and phylogenetic analysis of Sarcophagidae.

The Sarcophagidae family was considered as a crucial source of information involved in medical, hygienical and forensic investigations (Tomberlin and Benbow 2015; Ren et al. 2018). *Sarcophaga brevicornis* Ho, 1934, which belonged to the Sarcophagidae family and Diptera order, was mainly spread at Asia and usually found in decomposed carcasses and garbage (Sugiyama et al. 1988; Pérez-Moreno et al. 2006). The length of circular mitogenome was 15,152 bp (GenBank accession No. MK820720), which showed in a typical arthropod genome, including 13 protein-coding genes (13 PCGs), two ribosomal RNA (2 rRNA) genes, 22 transfer RNA (22 tRNA) genes, and an AT-rich region. Its nucleotide composition was A 39.0%, C 12.7%, G 10.6%, and T 37.7%.

The specimens were trapped in Beijing, China (39°26′N; 115°25′E) in May 2017. These species were identified by an expert according to traditional morphological approaches and then deposited in Guo’s lab (Changsha, Hunan, China) with a unique number (CSU19040902). Sequencing was performed in the Illumina HiSeq2500 platform after genomic DNA was isolated using the QIANamp Micro DNA Kit; *de novo* assembly and annotation were performed utilizing the MITObim V1.9 and SOAPdenovo v. 2.0 (Ren et al. 2019).

Phylogenetic trees of *S. brevicornis* and 11 published sarcophagid species were generated using neighbour-joining inference methods (NJ) based on the 13 PCGs, with *Calliphora vomitoria* and *Chrysomya pinguis* as outgroups (Figure 1). The tree showed that *S. brevicornis* was clearly separated from the other sarcophagid species, but it was closed to the species of *S. similis*. This study enriched the reference data of dipteran mitogenomes for species identification and evolution analysis of Sarcophagidae.

**Figure 1.**
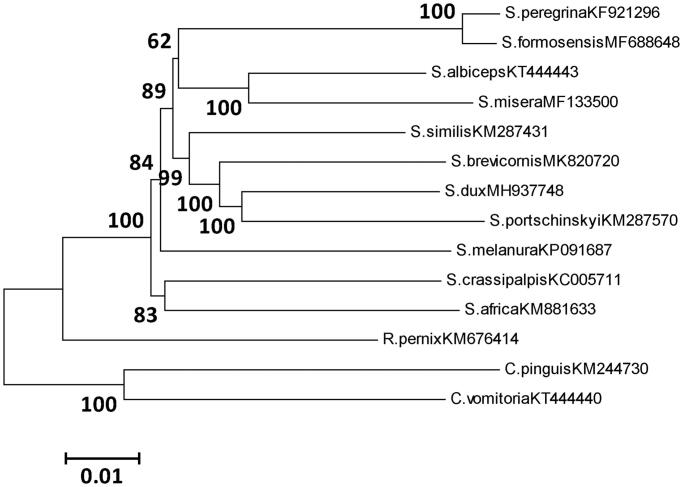
Phylogenetic analyses of 12 sarcophagid species were constructed using NJ method based on 13 PCGs. Morphological species identification and voucher ID were given in the label. Numbers on branches showed the bootstrap support value. The out-group consists of two specimens of Calliphora.

## References

[CIT0001] Pérez-Moreno S, Marcos-Garcia MA, Rojo S 2006 Comparative morphology of early stages of two Mediterranean Sarcophaga Meigen, 1826 (Diptera; Sarcophagidae) and a review of the feeding habits of Palaearctic species. Micron. 37:169–179. 16182548 10.1016/j.micron.2005.07.013

[CIT0002] Ren LP, Shang YJ, Chen W, Meng FM, Cai JF, Zhu GH, Chen LS, Wang Y, Deng JQ, Guo YD 2018 A brief review of forensically important flesh flies (Diptera: Sarcophagidae). Forensic Sci Res. 3:16–26. 30483648 10.1080/20961790.2018.1432099 PMC6197121

[CIT0003] Ren LP, Shang YJ, Yang L, Shen X, Chen W, Wang Y, Cai JF, Guo YD 2019 Comparative analysis of mitochondrial genomes among four species of muscid flies (Diptera: Muscidae) and its phylogenetic implications. Int J Biol Macromol. 127:357–364. 30658142 10.1016/j.ijbiomac.2019.01.063

[CIT0004] Sugiyama E, Shinonaga S, Kano R 1988 Sarcophagine flies from Nepal with the description of a new species (Diptera: Sarcophagidae). Med Entomol Zool. 39:355–362.

[CIT0005] Tomberlin JK, Benbow ME 2015 Forensic entomology: international dimensions and frontiers: CRC Press. Fla Entomol. 98:1015–1016.

